# Does Social Media Users’ Interaction Influence the Formation of Echo Chambers? Social Network Analysis Based on Vaccine Video Comments on YouTube

**DOI:** 10.3390/ijerph192315869

**Published:** 2022-11-29

**Authors:** Mingfei Sun, Xiaoyue Ma, Yudi Huo

**Affiliations:** School of Journalism and New Media, Xi’an Jiaotong University, Xi’an 710049, China

**Keywords:** health misinformation, echo chamber, user interaction, ERGM, COVID-19 vaccine

## Abstract

The characteristics and influence of the echo chamber effect (TECE) of health misinformation diffusion on social media have been investigated by researchers, but the formation mechanism of TECE needs to be explored specifically and deeply. This research focuses on the influence of users’ imitation, intergroup interaction, and reciprocity behavior on TECE based on the social contagion mechanism. A user comment–reply social network was constructed using the comments of a COVID-19 vaccine video on YouTube. The semantic similarity and Exponential Random Graph Model (ERGM) were used to calculate TECE and the effect of three interaction mechanisms on the echo chamber. The results show that there is a weak echo chamber effect (ECE) in the spread of misinformation about the COVID-19 vaccine. The imitation and intergroup interaction behavior are positively related to TECE. Reciprocity has no significant influence on TECE.

## 1. Introduction

With the rapid development of digital information technology, social media has become an important channel for the public to create, obtain, and share health information. Meanwhile, the diffusion content also becomes more diverse and plentiful due to the virtuality and openness of information spread channels, the extensity and anonymity of diffusion objects, and the decrease in communication costs. Users’ attention is attracted by a huge amount of mixed truth and misinformation without scrutinizing by the “gatekeeper” [1]. Simultaneously, user information acquisition is influenced by social media algorithms and cognitive biases, which results in selective exposure and sharing of information. It is common for users to interact with users who share their opinions, resulting in the echo chamber effect (TECE). It will generate the polarization of users’ attitudes on specific health agendas, further cause a split of group and society, exacerbate the spread of public panic, and threaten social stability [2,3]. For example, misinformation about COVID-19 caused public doubts about the security of vaccines, preventing the formation of the anti-epidemic coalition. The spread of genetically modified food misinformation has caused controversy, arousing public panic over GM foods.

Health misinformation diffusion and echo chambers have widely attracted researchers’ attention because of their negative effects. The research on misinformation diffusion paid more attention to the structure [4,5,6,7], influencing factors [8,9,10], the negative effect [11,12,13], and the misinformation rebuttal [14,15,16], etc. The research on TECE was detailed on political communication [17,18,19] and environmental protection [20,21], etc., which aimed to analyze the existence of echo chambers [22,23], the effect of the recommendation algorithm, and the influence of user cognition on echo chamber, etc. [24,25,26,27]. However, in the context of social media health misinformation diffusion, users are easily divided into different groups through contagion. However, there is limited research focused on the influence of user interaction on ECE during the diffusion of health misinformation.

Therefore, this research focuses on TECE on social media and explores the mechanism between user interaction and TECE. The research objectives are as follows. Firstly, this study aims to explore whether and to what extent ECE exists in the spread of health misinformation on social media platforms. Secondly, this study will analyze the influencing factors of ECE. User nodes constantly exchange opinions through forwarding, commenting, replying, and other behaviors during the diffusion of health misinformation. Thus, this study analyses the impact of different user behaviors on TECE under the contagion effect.

Specifically, a COVID-19 vaccine-related video on YouTube was used as a research case. The user interaction network was constructed based on user comment–reply data. Moreover, TECE was calculated by evaluating the homogeneity of users’ opinions. Meanwhile, the users’ interaction mechanism was subdivided, and the research hypotheses were proposed from the perspective of imitation, reciprocity, and intergroup interaction. This research integrated the user interaction network and TECE network, and Exponential Random Graph Model was used to calculate the influence of imitation, reciprocity, and intergroup interaction on TECE.

The significance of this research is mainly reflected in the following two aspects. Firstly, using the comment–reply social network, this study tested the presence of TECE in the spreading of health misinformation on YouTube, which complements earlier studies on Twitter, Weibo, and so on, that examined political, environmental, and other fields. [22,23]. Secondly, this research further explored the influence mechanism of TECE based on the echo chamber measurement result. In addition, the influencing factors were detailed based on social contagion theory. It is the in-depth exploration of users’ interactive behavior’s effect on group structure.

The organization of this article is as follows. Firstly, Section 2 reviews the literature from two aspects: TECE in misinformation diffusion and information contagion on social media. Secondly, Section 3 puts forward the hypothesis to explore the influence of users’ interaction on TECE. Section 4 explains the data collection, data processing, and measurement of variables. Section 5 verifies TECE on YouTube and the hypothesis by constructing two social networks. Based on the validation results, this paper is discussed in Section 6 and summarized in Section 7.

## 2. Literature Review

### 2.1. TECE of Misinformation

Misinformation is defined as information lacking evidence and violating known facts [28]. The disintermediation of social media provides an efficient path for the diffusion of much misinformation. As a consequence, the influencing factors of misinformation diffusion on social media cause wide attention. In terms of the content of misinformation, the type of information, the diffusion media, and the physiology of users have significant impacts on the spread of misinformation. In terms of expression, affective and comparative misinformation spread more easily than persuasive and uncertain misinformation, according to some studies [29]. Different persuasion strategies have different effects, and affective strategies have better effects on misinformation diffusion [10]. In terms of information type, the study of Zhou et al. showed that health warnings, advice, and effective support of misinformation spread rapidly on social media during the COVID-19 epidemic. In terms of communication media, media richness, and present formation influence audiences’ misinformation perception [8]. The research of Davidson and Kobayashi found that misinformation presentation media influenced users’ memory of content. Text and video would arouse users’ memory easily [30]. In terms of users’ psychology, different emotions such as confusion, hope, anxiety, and belief in information would influence users’ misinformation-sharing intention [31]. Ji et al.’s study showed that peer pressure in the group and individual social prestige would influence transgenesis-related misinformation diffusion [9].

Influenced by individual selective exposition, confirmation bias, and social contagion, social media users are prone to interact with users with similar attitudes and form a homogeneity group in the diffusion of misinformation. In the homogeneity group, users’ original opinion is reinforced incessantly which promotes the formation of echo chambers and opinion polarity [23]. Some researchers have explored TECE of social media misinformation diffusion. For example, some researchers analyzed TECE from the perspective of the topic and users. The results showed that there was homogeneity in the interactive network of GMO misinformation users, but there was also some interaction between pro- and anti-gene groups, which decreased the possibility of polarization [32,33]. It has been shown that TECE exists in social media misinformation diffusion by Choi et al. Users in echo chambers would spread misinformation more quickly and virally [34]. Bright et al. further pointed out that those anti-opinions would be undermined and marginalized in echo chambers, which limits the effect of damaging them [35]. Moreover, the echo chamber is related to the misinformation diffusion effect. When users are embedded into the hegemony group, they will process information by sharing value which will trigger and promote the formation of a collective narrative framework based on self-conformed [36]. As attitude polarization leads to complex contagion, hegemony and polarization can predict the cascading scale of information diffusion [37].

### 2.2. Information Contagion on Social Media

The spread of misinformation based on social media user interactions can be seen as a contagion of behavior and emotions. In biological science, contagion is a process that an individual can influence others in the group through direct or indirect contact [38]. Social media contagion research mostly focused on political selection, marketing, health communication, network events, etc., and has analyzed the influencing factors of contagion in political uncivil interactive behavior [39] or users’ comments in e-commerce [40].

Pan et al. thought that users’ social capital would influence the reciprocal behavior of health information [41]. Meanwhile, information characteristics influence users’ participation. For example, user engagement is positively affected by both positive and negative comments, whereas it is negatively affected by neutral comments [42]. The number of comments influences the contagion of users’ cyber-aggressive behavior and negative emotion [43]. In e-commerce, information quality such as comment–reply quantity, richness, and relatedness affect users’ purchase behavior [44]. The characteristics and locations of social media users influence the contagion of their interactive behavior [45]. Liang explored the influence of the cascade effect in information diffusion on social contagion [46]. The result showed that the depth of cascade and interaction negatively related to social contagion.

Social interaction is the bond of information transmission. Due to the bonding effect created by comments, replies, and postings, community structures can improve communication efficiency. Therefore, some researchers built a contagion model based on the contagion mechanism of user interaction. For example, Tsvetkova and Macy built an anti-contagion framework. They believed that the interaction between the instigator, the target, and the bystander constitutes the general reciprocity and the third-person effect in social contagion [47]. Song et al. thought that social media interactions will elevate individual opinions into collective regulation [48]. Hence, they proposed four contagion mechanisms. The results show that general reciprocity, direct reciprocity, leader mimicry, and peer mimicry would influence profanity contagion on social media. Based on mimicry and social interaction, Kwon and Gruzd analyzed the contagion effect of aggressive comments. The result showed that public swearing of root nodes could arouse peer swearing of children nodes [49]. Additionally, comments by a child node trigger a sequential curse. Chen and Wang pointed out that imitation, intergroup interaction, and reciprocity led to political incivility during the US presidential election with the spread of campaign mis-videos [39]. Besides, some research showed that information redundancy contributed to inferior social contagion within groups. Contrary to this theory, Harrigan et al.’s research on the influence of community structure on socially contagious behavior shows that social structure, especially reciprocal connection, determines the triadic structure of social behavior under continuous connection and interaction between community members [50]. Horsevad et al. further proved that network topology significantly affected the effectiveness of behavioral contagion [51].

### 2.3. Motivations

Through imitation, reciprocity, and intergroup interaction, users with similar views or emotions gather, and the original views are constantly amplified in the process of interaction and oscillation, creating an echo chamber [36]. The majority of existing studies on misinformation and echo chambers focus on political and marketing fields. They examine the factors contributing to misinformation and the presentation characteristics of echo chambers; however, they ignore the impact of behavior and emotional contagion on TECE due to interactions between users. Therefore, this research aims to analyze the influence of interactive contagion mechanisms on TECE from the perspective of user interaction during the diffusion of health misinformation on social media. The following questions will be further discussed.

RQ1: Is there an ECE in the spread of COVID-19-related misinformation through YouTube?

RQ2: Will TECE be influenced by the interaction mechanisms (imitation, reciprocity, and intergroup interaction) among users?

## 3. Hypotheses

Generally, information contagion on social media contains how users act toward others. As mis-videos spread on YouTube, users’ comments and replies build an interactive network. Within this network, the user’s comments will shape another user’s opinion which causes the contagion of misinformation and constructs a collective communication structure. Based on existing empirical research on information contagion summarized by this paper (Section 2.2), imitation, intergroup interaction, and reciprocity are closely related to information contagion. Therefore, the hypotheses are proposed around the influence of imitation, intergroup interaction, and reciprocity on TECE.

### 3.1. Imitation

Imitation is the synchronization of interpersonal affective and behavior by imitating others’ emotions and behaviors. Imitation contains verbal and non-verbal imitation. Non-verbal imitation includes imitation of facial emotion, movement, etc., and verbal imitation includes the imitation of writing style and language expression. On social media, users’ information communication or exposure arouses verbal or non-verbal imitation. For example, Kwon et al. included the third-party influence in the experimental study of the Facebook platform contagion phenomenon and believed that contact with social media information increased the possibility of users participating in social movements [52]. Tsvetkova and Macy believe that the third-person effect is a kind of imitation, and that observation can promote the adoption of observation behaviors [47]. Song et al. studied the contagion mechanism of political uncivilized information. The results showed that uncivilized information communication would cause political uncivilized behavior [48].

Communication adaptation theory suggests that the similarity of conversational styles helps to reduce social distancing and provide social support in user interaction. In online communities, a person strengthens the relationship with others by interacting with others, sharing knowledge, and imitating their behaviors, to maintain a positive social identity [50], integrate into the group, and form a high degree of cohesion [53]. In this process, individual behavior will evolve into collective behavior when the adoption behavior exceeds a threshold. Based on the above theory, misinformation exposure will influence individuals to express similar views and interact with each other, and this phenomenon will be strengthened in the process. Users who hold similar opinions will gather and arouse an ECE or polarization.

**H1.**  *In the process of health misinformation diffusion, users’ imitation behavior is positively related to TECE.*

### 3.2. Intergroup Interaction

Intergroup interaction refers to the interaction between group members who are separated due to differences in opinions, values, race, religion, gender, party affiliation, etc. Interaction between group members can be divided into intergroup interaction and intergroup contact, according to the interaction time. The research about intergroup interaction always analyzes unstructured and short time interaction between inter- and intra-group members, such as the influence of playing games and answering specific questions. The results through self-reported or physiological tests showed that intergroup interactions are negatively related to individual behaviors (such as collective anxiety), leading to intergroup bias and group separation. The study of MacInnis and Page-Gould shows that there is a threshold when group interactions transform into group contact by constructing a math model [54], and there are also some costs such as interactive anxiety and group bias. A positive group relationship will form when the contact threshold is exceeded. Stephan holds the view that anxiety released by intergroup interaction is negatively related to group members’ cognition, emotion, and behavior. At the cognitive level, it includes negative attitudes, stereotypes, prejudiced cognition of outgroups, etc. At the emotional level, it includes fear, threat, humiliation, hatred, etc. At the behavioral level, it includes avoidance and aggression by outgroup members [55]. Furthermore, the similarity between groups can increase bias, and users in different groups will present closer relationships compared with similar groups.

In the diffusion of social media misinformation, different users hold different views such as support, neutrality, and opposition due to differences in knowledge reserve and cognition, and they form different groups. Wang et al. indicated that users with different standpoints gathered into various groups and created echo chambers during misinformation diffusion [33]. Based on the de-individual effect in the extended social identity model and intergroup interaction theory, in a computer-mediated anonymous environment, users pay less attention to individual identity and shift their focus on group identity and regulation. Meanwhile, user interactions from different groups will lead to anxiety, prejudice, attack, avoidance, and other behaviors, which are not conducive to the formation of homogenous opinions. Therefore, this study hypothesized that:

**H2.**  *Intergroup interaction is positively related to TECE.*

### 3.3. Reciprocity

Reciprocity is a kind of dependency relationship which focuses on the information transmission ability among network nodes [56]. Atouba and Shumate indicate that reciprocity increases the depth of users’ relationships and can be used as a prerequisite [57]. It is possible to increase trust and make the exchange of information more effective through reciprocity. Song et al. divided reciprocity into general reciprocity and direct reciprocity in their research about cyber-aggressive behavior contagion [48]. General reciprocity refers to the negative effects generated by the user node’s perception of verbal attacks, which will produce a chain reaction of information or behavior contagion. Direct reciprocity refers to the interaction between information sender and receiver. The adoption of ideas and behaviors by individuals is the infectious result of direct interaction with infected nodes. Direct reciprocity widely appears in the social contagion model. For example, the independent cascade model assumes that direct exposure to infectious nodes will produce monotonic effects and further increase the likelihood of information and attitude infection. The liner threshold model indicates that attitude or behavior infection will appear when nodes adopt attitude or behavior beyond the tolerable threshold. The effective infection will occur in a reciprocal relationship. For example, users are prone to share emotions in the strongly connected network to strengthen the relationship.

Direct and frequent communication between nodes can promote the development of communities and increase the possibility of users joining the community. For example, Colleoni et al. found that users with reciprocity relationships display higher political homogeneity than those in non-reciprocity relationships [58]. Li et al. indicated that reciprocity could increase community cohesion in an online health community [59]. The research of Harrigan et al. showed that community structure, especially reciprocity, increased social contagion incessantly [50]. As with political and health information, reciprocity between users enhances the ability to transmit attitudes and opinions and facilitates the formation of homophily attitudes and echo chambers in the spread of misinformation on social media. So, this research proposed:

**H3.**  *Reciprocity significantly enhances TECE in the process of health misinformation propagation.*

## 4. Research Method

### 4.1. Data Collection

This research focuses on a mis-video about the COVID-19 vaccine, titled “Doctor’s Death After COVID Vaccine Is Being Investigated”, published on 20 January 2021, on YouTube. The 8 min, 20 s video, which analyzes the death of a Florida doctor who received a COVID-19 vaccine, has been viewed 5,627,885 times and sparked a discussion about COVID-19 vaccines in the comments section. This research used public API provided by YouTube to collect comments, users’ names for comments, and comment time about this video. Data were collected until 12 August 2022.

### 4.2. Data Process and Analyses

To calculate TECE, this research used the TF-IDF algorithm and cosine similarity to measure the homogeneity of users’ comments (Section 4.2.1). Furthermore, based on the result of homogeneity and comment–reply relationship, the social network of misinformation diffusion and the homogeneous user interaction network were constructed to calculate TECE (Section 4.2.2). Lastly, ERGM was used to measure variables and verify three hypotheses (Section 4.2.3).

#### 4.2.1. Measurement of Homogeneity

This research measures TECE by calculating the semantic similarity of user comments. The text similarity calculation regards the text as a collection of words. First, the proportion of a single word in a single user comment and a whole user comment is calculated, respectively. Based on the word frequency, the user review is transformed into a word vector.

In this study, the TF-IDF algorithm is used to calculate the word correlation in user comments set, and the calculation formula is as follows:

(1) $$tf_{i,j}=\frac{n_{i,j}}{\sum_{k}n_{k,j}}$$

(2) $$idf_{i}=\log\frac{\left|D\right|}{1+\left|\left\{j:t_{i}\epsilon d_{j}\right\}\right|}$$

(3) $$TF-IDF_{i,j}=tf_{i,j}\times idf_{i}$$

$n_{i,j}$ represents the frequency of $word_{i}$ in comment $d_{j}.$ $\sum_{k}n_{k,j}$ represents the frequency of all words in comment $d_{j}$. |D| represents the number of comments in corpus. $\left|\left\{j:t_{i}\epsilon d_{j}\right\}\right|$ represents the number of comments contains $word_{i}\;$[60].

The above algorithm is used to calculate the TF-IDF value of each word in each comment as its weight. Finally, the space vector of each comment is obtained.

*d* = (*t*_1_, *w*_1_; *t*_2_, *w*_2_; … …; *t_m_*, *w_m_*) (4)

$t_{i}$ represents the characteristic item of the comment, and $w_{i}$ represents the weight of the characteristic item.

Finally, the cosine similarity is used to calculate the similarity between comments, where A and B are the comment vectors to be compared, respectively. The calculation formula is as follows:

(5) $$\mathrm{Cos}\mathrm{ine}\;\mathrm{coefficient}=\cos\theta =\frac{A\cdot B}{∥A∥∥B∥}=\frac{\sum_{k=1}^{n}A_{k}B_{k}}{\sqrt{\sum_{k=1}^{n}A_{k}{}^{2}}\sqrt{\sum_{k=1}^{n}B_{k}{}^{2}}}$$

#### 4.2.2. Social Network Construction

Comments and replies under YouTube videos provide relational data sets for analyzing interactions between user nodes. Each YouTube user can comment on or reply to videos. The structure of user comments and replies is similar to that of threads. Threads start from the initial user comments, and other users can reply under the comments or use the @ symbol to reply to the comments. There are three situations: (1) User B replies to user A’s comments (B→A); (2) User C responds to user B by symbol @ (C→B); (3) Indirect and potential connection between user C and user A. Therefore, taking users as nodes and replies and comments as lines, the social network of misinformation diffusion and the homogeneous user interaction network are constructed based on the user interaction relationship and homogeneity measurement results, respectively [52].

#### 4.2.3. Variable Measurement and Hypothesis Testing

This research uses node characteristics in network indegree and outdegree to measure imitation behavior to test Hypothesis 1. This variable is continuous [39].

This study classifies user group identity based on comments. The user groups were divided into the COVID-19 vaccine support group, the opposition group, and the neutral group. Accordingly, user positions were divided into three types: vaccine neutrality, support, and opposition, which were coded as 0, 1, and 2, respectively. Two postgraduates independently coded the user’s position according to the user’s comments after training, and the reliability of the coding was calculated. The Kappa value was 1, indicating that the coding was highly reliable and results were acceptable. To test Hypothesis 2, this study measures user interactions with in-group or out-group (pro-vaccine or anti-vaccine) members using a homogeneous social network intergroup dynamics test. In the exponential random graph model, the interaction behavior between users was measured by “heterogeneous nodematch” [39].

The mutual index in an exponential random graph model is used to measure reciprocal interaction behavior between users in the directed network constructed by this study, i.e., user B replies to user A’s comments, and user A replies to user B’s replies (A→B and B→A) [39].

Overall hypothesis test uses the Exponential Random Graph model (ERGM). ERGM is a kind of statistical method based on relational data, which can be used to understand the influence of network member attributes or network node relations on the formation of the observed network by aggregating simple random graphs, binary independence models, and binary dependence models. In recent years, many scholars have used ERGM to study the relationship between node interaction mechanisms and the overall composition of the social network. Therefore, the ERGM package based on the Markov chain Monte Carlo simulation method in R software Statnet was used to fit the model. The influence of node indegree/outdegree (H1), node matching (H2), and reciprocity (H3) relationships on the formation of the final misinformation chamber network was explored in a directed network composed of user nodes and their comment relationships under the selected COVID-19 vaccine-related mis-video.

## 5. Results

Based on the method of Section 4.2, this research firstly constructs a misinformation diffusion network and homogeneous user interaction network, and the characteristics of those networks are described (Section 5.1). Subsequently, TECE of the COVID-19 vaccine-related mis-video is calculated (Section 5.2). Lastly, based on the misinformation diffusion network and homogeneous user interaction network (Section 5.1), this research uses ERGM to verify the influence of imitation, intergroup interaction, and reciprocity on TECE (Section 5.2).

### 5.1. Network Construction and Descriptive Analysis

This study used Python to collect comments, replies, and usernames under the selected videos. A total of 16,699 valid comments and replies were collected. The comment users with fewer than five replies were deleted to reduce the interference of discrete user nodes.

5691 comments and replies posted by 2991 users were deleted. The average user posted 1.90 comments or replies, with the highest number of replies being 260 out of 1520 commenting users.

**Figure 1:**
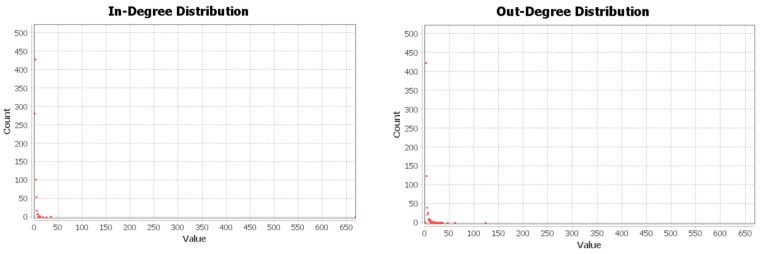
Distribution of in-degree and out-degree in the homogeneous user interaction network.

Firstly, a misinformation diffusion network was constructed (network 1) based on 5691 user comments and replies. This network has an average in-degree of 3.32, an average out-degree of 3.31, an average mutual of 0.07, and an average reciprocity of 61.65. Secondly, the homogeneity of comments was calculated, and the similarity results were normalized through TECE calculation method. Based on the similarity calculation results, the homogeneous user interaction network was constructed (network 2) with a total of 3190 connected edges. Homogenous user interaction (in-degree and out-degree distribution) is shown in Figure 1.

### 5.2. Measurement of TECE

**Figure 2:**
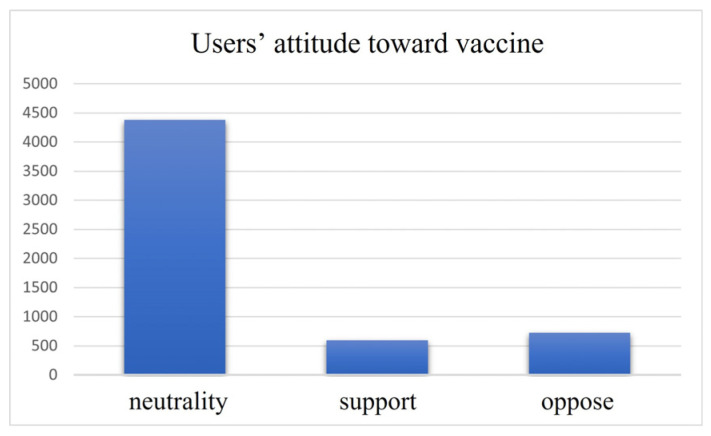
Distribution of users’ vaccine attitudes.

Users’ attitude was coded to understand users’ attitude toward the COVID-19 vaccine. As shown in Figure 2, the different label displays the heterogeneity of users’ attitude. As seen in the YouTube users’ information, most hold neutralizing attitudes, lacking any obvious support or opposition. COVID-19 vaccination is supported by 10.46% of the users, while it is strongly opposed by 12.67%.

**Figure 3:**
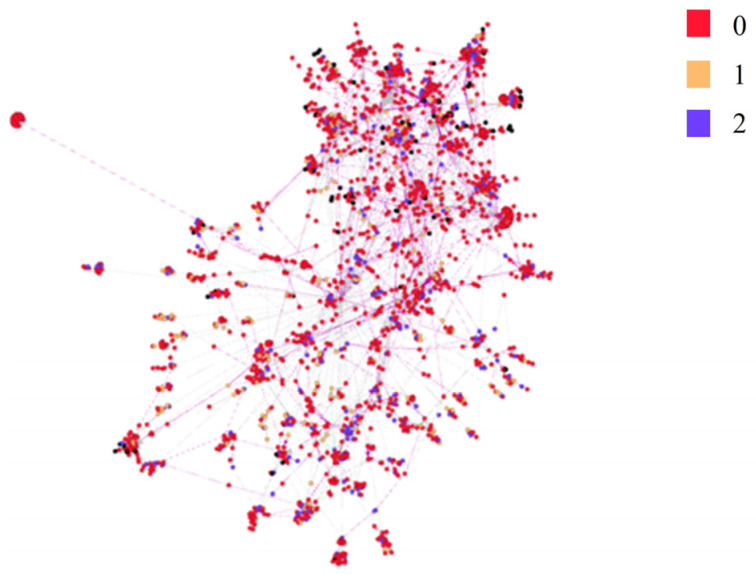
Echo chamber effect measurement. Note: The node color represents the user’s position, and the edge color intensity represents the difference in similarity intensity.

A user comment–reply social network was constructed. As shown in Figure 3, different node color represents different user standpoints (vaccine support, opposition, and neutrality). The average degree of this network is 1.723, and the average path length is 7.20, indicating there is a strong interaction between users. The similarity of users’ comments or replies was calculated to represent the homogeneity of users’ attitudes. The color of the edges in Figure 3 represents the homogeneity degree of users’ attitudes. The results indicate that users’ comment network shows significant modularity. The modularity coefficient is 0.844, and the number of communities is 120. Users’ standpoints are similar in the same module, and connections among modules are powerful. Clearly, users are used to interacting with nodes who hold similar viewpoints. In addition, interactions between in-group users increase their self-involvement, which promotes opinion polarization.

### 5.3. Hypothesis Test

This research used ERGM to test a hypothesis-based comment–reply network. The results are shown in Table 1. As stated in Hypothesis 1, imitation behavior is positively correlated with TECE during the diffusion of health misinformation. This research further details this hypothesis and holds that misinformation sending and receiving can promote TECE. The calculation result provides evidence for this supposition. In-degree (β=2.4918, *p* < 0.001) and out-degree (β=2.4940, *p* < 0.001) are positively related to node connection. Moreover, according to the estimated difference between in-degree and out-degree, posting misinformation makes it easier to trigger TECE.

Hypothesis 2 indicates that intergroup interaction is positively related to TECE. The result of empirical research shows that users who hold heterogeneous attitudes toward the COVID-19 vaccine accelerate TECE (β=0.0855, *p* < 0.001). Therefore, Hypothesis 2 is supported. The comments and replies among users of the three groups do not promote consensus on COVID-19 vaccines, but rather reinforce original views.

Hypothesis 3 considers that the reciprocal relationship constructed by users’ comments and replies increases TECE prominently. However, the results show that there is no significant relationship between reciprocity and TECE. Therefore, Hypothesis 3 is refused.

## 6. Discussion

### 6.1. Echo Chamber Effect in Health Misinformation Diffusion

TECE was examined in this study through an examination of the comments on YouTube related to the COVID-19 vaccine. In the results, social media users generally communicated with users of the same position, and most interactions took place among vaccine supporters or opponents, resulting in communities with homogenous views. Nevertheless, data analysis showed that the interactive network contained cross-stance user interactions. It indicates that a weak ECE is found in this study, and heterogeneous user interactions still exist within heterogeneous user interactions, which supports Wang and Song’s research conclusion [32]. ECE has been found in the discussion of GM rumors on the Weibo platform, as well as a great deal of interaction between pro- and anti-GMO communities, which may reduce the possibility of opinion polarization [32]. However, the conclusion of this study is different from some existing studies. For example, Cinelli et al. and Cota et al. show that there is a strong division between different groups under the three topics of gun control, vaccines, and abortion on Facebook and Twitter. Homogenous groups dominate online interaction [22,23]. The occurrence of this phenomenon may be related to the characteristics of the YouTube platform. There are strong connections in user association networks on Facebook and Twitter. However, YouTube does not form a homogenous cluster of user interaction based on strong connections. As a public space for video viewing, most users only view the video or talk about the content and pay less attention to others. It is still difficult to form homogeneous groups despite some users’ shared interests.

### 6.2. Influence of User Interaction on TECE of Health Misinformation

Social media provides an anonymous and convenient information production and communication platform. Users with different views interact and communicate after health misinformation is posted. In this process, selective exposure and attitudinal collision of users promote the formation of homogeneous communities and echo chambers. This research took a COVID-19 vaccine-related mis-video as an example and explored the influence of user interaction (imitation, intergroup interaction, and reciprocity) on TECE.

First, the result based on ERGM showed that imitation promoted TECE of health misinformation, and imitation has the strongest effect on TECE among the three interaction mechanisms. This conclusion is consistent with the research of Song et al. [48] In the study of the influence of four infectious mechanisms (generalized reciprocity, direct reciprocity, leadership imitation, and peer imitation) on the infection of political swear words, they found that peer imitation made the greatest contribution to the infection under the influence of social learning effect, followed by universal reciprocity and direct imitation. This research extends this conclusion to TECE of health misinformation diffusion. Different from offline information diffusion, online discourse is multiple and synchronous. Users can view others’ attitudes in time, imitate others’ dialogic style, and observe the discourse interaction. Ultimately, the spiraling collective discourse culture is formed through the learning and transformation of discourse content.

Second, the result showed that intergroup interaction positively affected TECE of health misinformation. This conclusion is similar to the study of Chen and Wang [39]. During the 2020 US presidential election, the interaction between groups under mis-campaign videos contributed to the contagion of political incivility. This conclusion is extended to the health communication research field. By clustering homogenous groups, users who approve or oppose vaccines promote group opinion polarization. Intergroup interaction, however, has a limited effect due to YouTube’s characteristics. When users watch videos on YouTube, their learning attitudes take center stage. This behavior is different from single information production. Users are more likely to be persuaded and accept others’ attitudes when communicating with people from different standpoints.

Third, this research supposes that reciprocity is positively related to TECE, which is conducive to the formation of homogeneous attitudes. However, this hypothesis was not supported. This conclusion is different from existing studies [46,61]. For example, when people express political opinions, swearing tends to happen between two users in the HKGolden forum [48]. In online MOOC forum, the reciprocity relation promotes the transfer of knowledge [62]. Based on the empirical results of this study, frequent public opinion expression can promote the agglomeration of homogeneous groups and echo of views, but direct private opinion exchange fails to reach a consensus among users. The occurrence of this phenomenon may be related to the following reasons. On the one hand, information is exposed widely in communities due to the imitation effect. Users will be more likely to obey the norm and transmit similar opinions when they perceive it as being a group regulation [63]. However, under reciprocity, independent communication between users does not result in perceived group norms. It is also not conducive to accepting others’ opinions when arguments have not been fully discussed. On the other hand, users present themselves on social media, and their viewpoints represent personal identity. Face-to-face communication with users arouses their defensive psychology and perceived face threat, which makes it difficult to reach a consensus [64].

### 6.3. Implications

This research explored TECE and its formation mechanism systematically in COVID-19 vaccine-related misinformation diffusion on YouTube. It has significant theoretical and practical value.

In theory, firstly, different from constructing a user interactive network through a reply relationship, this research focuses on the comment–reply relationship and content and analysis TECE of the interactive network. This conclusion extends the research of TECE on Twitter or Weibo to the video platform and demonstrates the possibility of echo chamber research on the video platform. Secondly, this research further explored the influence of imitation, intergroup interaction, and reciprocity on TECE in misinformation diffusion based on social contagion theory from the perspective of users’ interactive mechanisms. Most research on ECE analyzes whether echo chambers exist and those effects but ignore the influence of user interaction on ECE. This research is not only an extension and supplement to the formation mechanism of ECE, but also extends the research view to YouTube to verify and explain the difference of users’ interaction effect.

In practice, the results of this study provide guidance to relevant government departments on how to effectively manage public health information through social media. Firstly, the research indicates that there is a weak ECE on COVID-19 vaccine videos on YouTube. The intergroup and intragroup interactive behavior coexisted. Therefore, during public health crises, government departments should attach importance to the management of social media platforms. Relevant departments should take use of video platforms to release authority and comprehensive health information quickly to stymie the formation of TECE of health misinformation and users’ misbelief or incorrect health behaviors. Secondly, the result shows that imitation and intergroup interaction positively influence the formation of health misinformation echo chambers. Therefore, from the perspective of imitation, the government and social media should pay much attention to users’ health belief which has high in-degree and out-degree. Government can monitor and manage those users to prevent the health misinformation echo chamber by providing health knowledge training and improving their health literacy to promote the diffusion and imitation of scientific health information. From the perspective of intergroup interaction, the formation of users’ misbeliefs can be stymied by improving the social media information recommendation algorithm which will make users receive diversified health information and opinions.

### 6.4. Limitations and Future Directions

There are also some limitations. First of all, this research only considers three different social interaction patterns when calculating the influence of users’ interaction on TECE. Considering other interaction factors such as indirect reciprocity and diffusion cascade can enrich the underlying mechanism between users’ interaction and ECE. Secondly, the coding of the user’s position is based on the content posted in the past, which deviates from the real user’s position to some extent. Therefore, future research can integrate a questionnaire method to measure users’ standpoints. Finally, influenced by the characteristics of YouTube, users always discuss a specific video and form a virtual community, which is different from other social media platforms such as Twitter and Weibo. Therefore, this research merely focused on one COVID-19 vaccine-related video to explore the formation mechanism of TECE. Future research will select more videos to verify the conclusion further.

## 7. Conclusions

This research focused on the mechanism between users’ interaction and TECE in the context of misinformation diffusion on social media. A COVID-19 vaccine-related mis-video on YouTube was taken as an example, and an interactive network was constructed based on the comment–reply relationship to calculate TECE. ERGM was used to analyze the influence of imitation, intergroup interaction, and reciprocity on TECE. The results showed that there was a weak ECE on YouTube, while imitation and intergroup interaction had a positive effect on TECE. The results provide a meaningful inspiration for government to use video platforms to eliminate the negative impact of health misinformation and improve public health literacy.

## Figures and Tables

**Table 1:** ERGM results.

	Estimate	SE	Z Value
edge	−5.8586 ***	0.0273	−214.621
In-degree	2.4918 ***	0.0730	34.119
Out-degree	2.4940 ***	0.0742	33.630
Intergroup interaction	0.0855 ***	0.0250	3.415
reciprocity	60.4668	0.0436	0.093

Note: ***, *p* < 0.001.

## Data Availability

The data presented in this study are available on request from the corresponding author. The data are not publicly available due to the privacy of participants.

## References

[1] Lu B., Sun J., Chen B., Wang Q., Tan Q. (2022). A Study on the Effectiveness of Rumor Control via Social Media Networks to Alleviate Public Panic About COVID-19. Front. Public Health.

[2] Osman M., Adams Z., Meder B., Bechlivanidis C., Verduga O., Strong C. (2022). People’s understanding of the concept of misinformation. J. Risk Res..

[3] Compton J., Linden S., Cook J., Basol M. (2021). Inoculation theory in the post-truth era: Extant findings and new frontiers for contested science, misinformation, and conspiracy theories. Soc. Personal. Psychol. Compass.

[4] Ceron W., Gruszynski Sanseverino G., De-Lima-Santos M., Quiles M.G. (2021). COVID-19 fake news diffusion across Latin America. Soc. Netw. Anal. Min..

[5] Ruan Z., Yu B., Shu X., Zhang Q., Xuan Q. (2020). The impact of malicious nodes on the spreading of false information. Chaos.

[6] Vosoughi S., Roy D., Aral S. (2018). The spread of true and false news online. Science.

[7] Shin J., Jian L., Driscoll K., Bar F. (2017). Political rumoring on Twitter during the 2012 US presidential election: Rumor diffusion and correction. New Media Soc..

[8] Zhou C., Li K., Lu Y. (2021). Linguistic characteristics and the dissemination of misinformation in social media: The moderating effect of information richness. Inf. Processing Manag..

[9] Ji J., Chao N., Ding J. (2019). Rumormongering of genetically modified (GM) food on Chinese social network. Telemat. Inform..

[10] Chen S., Xiao L., Mao J. (2021). Persuasion strategies of misinformation-containing posts in the social media. Inf. Processing Manag..

[11] Chen Y.P., Chen Y.Y., Yang K.C., Lai F., Huang C.H., Chen Y.N., Tu Y.C. (2022). The Prevalence and Impact of Fake News on COVID-19 Vaccination in Taiwan: Retrospective Study of Digital Media. J. Med. Internet Res..

[12] Li P.Y., Chen B., Deveaux G., Luo Y., Tao W., Li W., Wen J., Zheng Y. (2022). Cross-Verification of COVID-19 Information Obtained From Unofficial Social Media Accounts and Associated Changes in Health Behaviors: Web-Based Questionnaire Study Among Chinese Netizens. JMIR Public Health Surveill..

[13] Moravec P.L., Minas R.K., Dennis A.R. (2019). Fake news on social media: People believe what they want to believe when it makes no sense at all. MIS Q..

[14] Burel G., Farrell T., Alani H. (2021). Demographics and topics impact on the co-spread of COVID-19 misinformation and fact-checks on Twitter. Inf. Processing Manag..

[15] King K.K., Wang B., Escobari D., Oraby T. (2021). Dynamic Effects of Falsehoods and Corrections on Social Media: A Theoretical Modeling and Empirical Evidence. J. Manag. Inform. Syst..

[16] Chung M., Kim N. (2021). When I Learn the News is False: How Fact-Checking Information Stems the Spread of Fake News Via Third-Person Perception. Hum. Commun. Res..

[17] Dubois E., Blank G. (2018). The echo chamber is overstated: The moderating effect of political interest and diverse media. Inf. Commun. Soc..

[18] Wollebæk D., Karlsen R., Steen-Johnsen K., Enjolras B. (2019). Anger, Fear, and Echo Chambers: The Emotional Basis for Online Behavior. Soc. Media+ Soc..

[19] Han J., Lee Y., Lee J., Cha M. (2022). News comment sections and online echo chambers: The ideological alignment between partisan news stories and their user comments. Journalism.

[20] Miller A., Arndt S., Engel L., Boot N. (2021). Nature conservation in a digitalized world: Echo chambers and filter bubbles. Ecol. Soc..

[21] Williams H.T.P., McMurray J.R., Kurz T., Hugo Lambert F. (2015). Network analysis reveals open forums and echo chambers in social media discussions of climate change. Glob. Environ..

[22] Cota W., Ferreira S.C., Pastor-Satorras R., Starnini M. (2019). Quantifying echo chamber effects in information spreading over political communication networks. Epj. Data Sci..

[23] Cinelli M., Morales G.D.F., Galeazzi A., Quattrociocchi W., Starnini M. (2021). The echo chamber effect on social media. Proc. Natl. Acad. Sci. USA.

[24] Cardenal A.S., Aguilar-Paredes C., Cristancho C., Majó-Vázquez S. (2019). Echo-chambers in online news consumption: Evidence from survey and navigation data in Spain. Eur. J. Commun..

[25] Rhodes S.C. (2022). Filter Bubbles, Echo Chambers, and Fake News How Social Media Conditions Individuals to Be Less Critical of Political Misinformation. Polit. Commun..

[26] Geschke D., Lorenz J., Holtz P. (2019). The triple-filter bubble: Using agent-based modelling to test a meta-theoretical framework for the emergence of filter bubbles and echo chambers. Br. J. Soc. Psychol..

[27] Bessi A. (2016). Personality Traits and Echo Chambers on Facebook. Comput. Hum. Behav..

[28] Bode L., Vraga E.K. (2015). In Related News, That Was Wrong: The Correction of Misinformation Through Related Stories Functionality in Social Media. J. Commun..

[29] Zhou C., Xiu H., Wang Y., Yu X. (2021). Characterizing the dissemination of misinformation on social media in health emergencies: An empirical study based on COVID-19. Inf. Processing Manag..

[30] Davidson B.M., Kobayashi T. (2022). The effect of message modality on memory for political disinformation: Lessons from the 2021 U.S capitol riots. Comput. Hum. Behav..

[31] Lu X., Vijaykumar S., Jin Y., Rogerson D. (2022). Think before you Share: Beliefs and emotions that shaped COVID-19 (Mis)information vetting and sharing intentions among WhatsApp users in the United Kingdom. Telemat. Inform..

[32] Wang X.H., Song Y.Y. (2020). Viral misinformation and echo chambers: The diffusion of rumors about genetically modified organisms on social media. Internet Res..

[33] Wang D., Qian Y. (2021). Echo Chamber Effect in Rumor Rebuttal Discussions About COVID-19 in China: Social Media Content and Network Analysis Study. J. Med. Internet Res..

[34] Choi D., Chun S., Oh H., Han J., Kwon T. (2020). Rumor Propagation is Amplified by Echo Chambers in Social Media. Sci. Rep..

[35] Bright J., Marchal N., Ganesh B., Rudinac S. (2022). How Do Individuals in a Radical Echo Chamber React to Opposing Views? Evidence from a Content Analysis of Stormfront. Hum. Commun. Res..

[36] Del Vicario M., Vivaldo G., Bessi A., Zollo F., Scala A., Caldarelli G., Quattrociocchi W. (2016). Echo chambers: Emotional contagion and group polarization on facebook. Sci. Rep..

[37] Törnberg P. (2018). Echo chambers and viral misinformation: Modeling fake news as complex contagion. PLoS ONE.

[38] Dodds P.S., Watts D.J. (2005). A generalized model of social and biological contagion. J. Theor. Biol..

[39] Chen Y., Wang L. (2022). Misleading political advertising fuels incivility online: A social network analysis of 2020 U.S. presidential election campaign video comments on YouTube. Comput. Hum. Behav..

[40] Khobzi H., Lau R.Y.K., Cheung T.C.H. (2019). The outcome of online social interactions on Facebook pages. Internet Res..

[41] Pan W., Shen C., Feng B. (2017). You Get What You Give: Understanding Reply Reciprocity and Social Capital in Online Health Support Forums. J. Health Commun..

[42] Kramer A.D.I., Guillory J.E., Hancock J.T. (2014). Experimental evidence of massive-scale emotional contagion through social networks. Proc. Natl. Acad. Sci. USA.

[43] Petit J., Li C., Ali K. (2021). Fewer people, more flames: How pre-existing beliefs and volume of negative comments impact online news readers’verbal aggression. Telemat. Inform..

[44] Kim D., Park S., Yi S. (2021). Relevant and rich interactivity under uncertainty: Guest reviews, host responses, and guest purchase intention on Airbnb. Telemat. Inform..

[45] Xu S., Luttman S. (2021). Networked publics in# NoDAPL protests: Interactions among activist publics and influence of locality and proximity on socially mediated networks. New Media Soc..

[46] Liang H. (2021). Decreasing social contagion effects in diffusion cascades: Modeling message spreading on social media. Telemat. Inform..

[47] Tsvetkova M., Macy M.W. (2014). The social contagion of generosity. PLoS ONE.

[48] Song Y., Lin Q., Kwon K.H., Choy C.H., Xu R. (2022). Contagion of offensive speech online: An interactional analysis of political swearing. Comput. Hum. Behav..

[49] Kwon K.H., Gruzd A. (2017). Is offensive commenting contagious online? Examining public vs interpersonal swearing in response to Donald Trump’s YouTube campaign videos. Internet Res..

[50] Harrigan N., Achananuparp P., Lim E. (2012). Influentials, novelty, and social contagion. Soc. Netw..

[51] Horsevad N., Mateo D., Kooij R.E., Barrat A., Bouffanais R. (2022). Transition from simple to complex contagion in collective decision-making. Nat. Commun..

[52] Kwon K.H., Stefanone M.A., Barnett G.A. (2014). Social network influence on online behavioral choices: Exploring group formation on social network sites. Am. Behav. Sci..

[53] Welbers K., de Nooy W. (2014). Stylistic Accommodation on an Internet Forum as Bonding. Am. Behav. Sci..

[54] MacInnis C.C., Page-Gould E. (2015). How Can Intergroup Interaction Be Bad If Intergroup Contact Is Good? Exploring and Reconciling an Apparent Paradox in the Science of Intergroup Relations. Perspect. Psychol. Sci..

[55] Stephan W.G. (2014). Intergroup anxiety: Theory, research, and practice. Pers. Soc. Psychol. Rev..

[56] Saffer A.J., Pilny A., Sommerfeldt E.J. (2022). What Influences Relationship Formation in a Global Civil Society Network? An Examination of Valued Multiplex Relations. Commun. Res..

[57] Atouba Y., Shumate M. (2010). Interorganizational Networking Patterns Among Development Organizations. J. Commun..

[58] Colleoni E., Rozza A., Arvidsson A. (2014). Echo Chamber or Public Sphere? Predicting Political Orientation and Measuring Political Homophily in Twitter Using Big Data. J. Commun..

[59] Li H., Kraut R.E., Zhu H. (2021). Technical Features of Asynchronous and Synchronous Community Platforms and their Effects on Community Cohesion: A Comparative Study of Forum-based and Chat-based Online Mental Health Communities. J. Comput.-Mediat. Commun..

[60] Yang S., Yao J., Qazi A. (2020). Does the review deserve more helpfulness when its title resembles the content? Locating helpful reviews by text mining. Inf. Processing Manag..

[61] Qiao T., Shan W., Zhang M., Liu C. (2019). How to facilitate knowledge diffusion in complex networks: The roles of network structure, knowledge role distribution and selection rule. Int. J. Inf. Manag..

[62] Wu B., Wu C.C. (2021). Research on the mechanism of knowledge diffusion in the MOOC learning forum using ERGMs. Comput. Educ..

[63] Rui J.R., Cui X. (2022). How technological affordances predict political expression via Quora: Mediated by risk appraisal and moderated by social motivation. Cyberpsychology.

[64] Malloch Y., Feng B. (2021). What You Say and Where You Say It Matter: Effects of Facebook Message Publicity and Support Type on Evaluation of Support Message Quality. Soc. Sci. Comput. Rev..

